# NAP1L1 interacts with hepatoma-derived growth factor to recruit c-Jun inducing breast cancer growth

**DOI:** 10.1186/s12935-021-02301-3

**Published:** 2021-11-13

**Authors:** Shu Liu, Yewei Zhang, Shien Cui, Dajiang Song, Bo Li, Qian Chen, Guangyu Yao, Bin Gong

**Affiliations:** 1 Department of Breast Surgery, The Affiliated Hospital of Guizhou Medical University, Guiyang, 550001 Guizhou People’s Republic of China; 2 Guizhou Medical University, Guiyang, Guizhou China; 3 Breast Center, Department of General Surgery, Nanfang Hospital Southern Medical University, Guangzhou, China; 4 Breast Center, Department of General Surgery, Zhongshan City People’s Hospital, Zhongshan, Guangzhou, China; 5 Cancer Center, Integrated Hospital of Traditional Chinese Medicine, Southern Medical University, Guangzhou, China; 6 Department of Oncology Plastic Surgery, Hunan Province Cancer Hospital and The Affiliated Cancer Hospital of Xiangya School of Medicine, Central South University, Changsha, Hunan China

**Keywords:** Breast cancer, Oncogene, NAP1L1, HDGF, C-JUN

## Abstract

**Background:**

Breast cancer is a common cancer among women in the world. However, its pathogenesis is still to be determined. The role and molecular mechanism of Nucleosome Assembly Protein 1 Like 1 (NAP1L1) in breast cancer have not been reported. Elucidation of molecular mechanism might provide a novel therapeutic target for breast cancer treatment.

**Methods:**

A bioinformatics analysis was conducted to determine the differential expression of NAP1L1 in breast cancer and find the potential biomarker that interacts with NAP1L1 and hepatoma-derived growth factor (HDGF). The expression of NAP1L1 in tissues was detected by using immunohistochemistry. Breast cancer cells were transfected with the corresponding lentiviral particles and siRNA. The efficiency of transfection was measured by RT-qPCR and western blotting. Then, MTT, Edu, plate clone formation, and subcutaneous tumorigenesis in nude mice were used to detect the cell proliferation in breast cancer. Furthermore, coimmunoprecipitation (Co-IP) assay and confocal microscopy were performed to explore the detailed molecular mechanism of NAP1L1 in breast cancer.

**Results:**

In this study, NAP1L1 protein was upregulated based on the Clinical Proteomic Tumor Analysis Consortium (CPTAC) database. Consistent with the prediction, immunohistochemistry staining showed that NAP1L1 protein expression was significantly increased in breast cancer tissues. Its elevated expression was an unfavorable factor for breast cancer clinical progression and poor prognosis. Stably or transiently knocking down NAP1L1 reduced the cell growth in vivo and in vitro via repressing the cell cycle signal in breast cancer. Furthermore, the molecular basis of NAP1L1-induced cell cycle signal was further studied. NAP1L1 interacted with the HDGF, an oncogenic factor for tumors, and the latter subsequently recruited the key oncogenic transcription factor c-Jun, which finally induced the expression of cell cycle promoter Cyclin D1(CCND1) and thus the cell growth of breast cancer.

**Conclusions:**

Our data demonstrated that NAP1L1 functions as a potential oncogene via interacting with HDGF to recruit c-Jun in breast cancer.

**Supplementary Information:**

The online version contains supplementary material available at 10.1186/s12935-021-02301-3.

## Introduction

Breast cancer is a serious disease in which malignant cells are formed in breast tissues. It is the second most common cancer in women after skin cancer. The etiology of breast cancer is still unclear, which may be related to the patient’s age, family history, hormone, long-term excessive drinking, and carrying mutation genes related to breast cancer. These factors alone or together induce the abnormal expression of some genes [1–6] and thus promote the pathogenesis of breast cancer.

Nucleosome Assembly Proteins (NAPs) belong to a highly conserved family of histone chaperones present in animals, plants, and yeast. They are crucial for the shuttling and incorporation of histones into nucleosomes and participate in the assembly and disassembly of nucleosomes, thus contributing to chromatin structure organization [7]. The NAP family comprises NAP1L1,2,3,4,5 and 6. In these genes, NAP1L1 is more widely reported to participate in the pathogenesis of tumors. It is preliminarily found as the human homolog of the yeast NAP-1 protein and promotes the cumulative nucleosome formation [8]. The increased expression of NAP1L1 can distinguish the grade of glioma and malignant behavior of small intestinal carcinoid [9]. In addition, elevated NAP1L1 level was shown as potential diagnostic and unfavorable prognostic biomarkers for some tumors and stimulated tumor progression and doxorubicin chemotherapy resistance in tumors including colorectal cancer [10, 11], renal cancer [12], liver cancer [13–15], glioblastoma [16] and pancreatic neuroendocrine neoplasm [17]. These data demonstrate the significance of NAP1L1 in tumor pathogenesis. Yet, the role and molecular mechanism of NAP1L1 in breast cancer have not been reported.

Here, NAP1L1 protein was found to be upregulated and considered as an unfavorable factor for the poor progression and prognosis of breast cancer patients. Furthermore, NAP1L1 was observed to be an interaction factor of HDGF, recruiting the key oncogenic transcription factor c-Jun and thus inducing the expression of cell cycle promoter CCND1, which finally stimulated breast cancer proliferation. These detailed data indicate NAP1L1 as a potential oncogene, significantly participating in the pathogenesis of breast cancer.

## Materials and methods

### Bioinformatics assay

BIOGRID web (https://thebiogrid.org/) was used to find the potential biomarker interaction with NAP1L1 and HDGF. UALCAN web (http://ualcan.path.uab.EdU/) was used to analyze the differential protein expression of NAP1L1 in breast cancer based on the analysis of Clinical Proteomic Tumor Analysis Consortium (CPTAC) database.

### Cell culture

Two breast cancer cell lines (MCF-7 and MDA-MB-231) were obtained from the Cell Bank of the Chinese Academy of Science (Shanghai, China). MDA-MB-231 cell lines were cultured in Dulbecco’s modified Eagle medium (DMEM) (PAN-Biotech, Aidenbach, Germany). MCF-7 cell lines were cultured in Roswell Park Memorial Institute-1640 (RPMI-1640) (PAN-Biotech, Aidenbach, Germany). All cell lines were incubated in a 5% CO_2_ humidified chamber at 37 ℃ and supplemented with 10% fetal bovine serum (FBS; PAN-Biotech, Aidenbach, Germany).

### Immunohistochemistry

Breast cancer tissue microarray (TMA) was purchased from Shanghai Outdo Biotech (Shanghai Outdo Biotech, Shanghai, China). Among patients who had undergone surgical resection for breast cancer between January 2001 and August 2004, 97 patients (from 29 to 83 years old, with a mean age of 51 years) were selected for the construction of TMA. All the samples of primary invasive breast cancer received no treatment prior to surgery. They were used to evaluate the NAP1L1 protein expression. Consent from the patients and approval from the Ethics Committee of Shanghai Outdo Biotech were obtained before using the clinical samples for research purposes. Tissue sections from the in vivo experiments were used to detect Ki67 and proliferating cell nuclear antigen (PCNA) protein expression levels using immunohistochemistry. The antibodies used were rabbit anti-PCNA (1:200; Proteintech, Rosemont, USA) and mouse anti-Ki67 (1:200; Signalway Antibody, Maryland, USA) (Additional file 4: Table S3). The indirect streptavidin-peroxidase method was used according to the manufacturer’s standard experiment guidelines. Cell staining was respectively scored by two pathologists blinded to the clinical parameters. The extent of staining, defined as the percentage of positively stained tumor cells in relation to the whole tissue area, was scored on a scale of 0–4 as follows: 0, < 10%; 1, 10–25%; 2, 26–50%; 3, 50–75%; and 4, > 75%. The staining intensity was scored as 0–3 (Negative: 0; Weak expression: 1; Positive expression: 2; Strong expression: 3. The score represents the product of the positive staining score, and the color intensity score was used as the final staining score for NAP1L1, Ki-67, and PCNA (0–12). For statistical analysis, final staining scores of 0–6 and 8–12 were considered to show low and high expression, respectively.

### RT-PCR and QPCR

Total RNA was isolated from breast cancer cells using Trizol reagent (Invitrogen, America), and cDNA was synthesized using reverse transcription reagents (TaKaRa Bio, Shiga, Japan). The parameters were set up as follows: 37 ℃ for 15 min, 85 ℃ for 40 s, and 4 ℃. Furthermore, cDNA was used as a template for amplification using specific primers (Additional file 2: Table S1). Bio-Rad T100 and Bio-Rad CFX96 detection systems were applied for RT-PCR and QPCR, respectively, following the manufacturer’s instructions. The parameters were set as follows: 95 °C for 30 s, 40 cycles of 95 °C for 5 s, 60 °C for 30 s, and dissociation stage. The relative gene expression levels were calculated using the 2^−ΔΔCt^ method. Beta-actin was used as an internal control.

### Lentivirus infection

Lentiviral particles carrying the ShRNA-NAP1L1 precursor were purchased from GeneChem (Shanghai, China) (Additional file 3: Table S2). Twelve hours before infection, the breast cancer cells (5 × 10^4^/well) were seeded into 24-well plates. MCF-7 and MDA-MB-231 cells were transfected with lentiviral particles harboring experimental or control vectors. Lentivirus (volume = multiplicity of infection × number of cells/lentivirus titer) mixed with 5 μg/mL polybrene was added to cells. After 12 h of culture, the cells were replaced with RPMI-1640 or DMEM containing 10% FBS. The medium was replaced with a fresh medium containing 5 μg/mL puromycin (MedChemExpress, Shanghai, China) after 7 days of infection. Next, this medium containing puromycin was replaced every 2 days until the control cells were completely dead. Green fluorescent protein (GFP) was used as a marker to monitor the infection efficiency. Subsequent experiments were carried out until the virus transduction efficiency via GFP expression was above 90%. The silencing efficiency for NAP1L1 was tested by RT-qPCR and Western blot analysis.

### SiRNA and plasmid transfection

SiRNAs for NAP1L1 were designed and synthesized by RiboBio (Guangzhou, China) (Additional file 3: Table S2). Plasmids for HDGF and c-Jun were obtained from Vigene Biosciences. Twelve hours before transfection, the breast cancer cells were plated into 6-well plates (Nest Biotech, China) and cultured to 30–50% confluence. SiRNA and scrambled siRNA were transfected at a concentration of 50 nM, pcDNA3.1-HDGF overexpression plasmid and pcDNA3.1-c-Jun overexpression plasmid and respective control plasmids were transfected using Lipofectamine 3000 Transfection Reagent (Invitrogen, Carlsbad, CA, USA) according to the manufacturer’s protocol. Forty-eight to 72 h later, the cells were collected for further experiments.

### MTT assay

The breast cancer cells (2000/well) were seeded into 96-well plates. For lentivirus-mediated shNAP1L1 expression, the cells were incubated for a week. For transient transfections with si-NAP1L1, the cells were cultured for four days. Subsequently, 20 μL of MTT (5 μg/μL in PBS) (Sigma, St Louis, MO) solution was added to each well and incubated for 4 h. Then, the formazan crystals formed by viable cells were solubilized in 150 mL dimethyl sulfoxide (Sigma, St Louis, MO), and the absorbance (OD) was measured at 490 nm. All the experiments were repeated at least three times.

### Plate clone formation

Clone formation was studied following our previous study. The cells were seeded in 6-well culture plates at 500 cells/well. After incubation for 14 days, the cells were washed twice with D-Hanks solution and stained with hematoxylin solution. The number of colonies was counted under a microscope. All experiments were performed at least three times.

### Edu staining

For the Edu incorporation assay, the proliferating breast cancer cells were examined using a Cell-Light Edu Apollo 488 or 567 In Vitro Imaging Kit (RiboBio) following the manufacturer’s protocol. The breast cancer cells (8000/well) were seeded into 96-well plates. After incubation with 10 mM Edu for 2 h, the breast cancer cells were fixed with 4% paraformaldehyde, permeabilized in 0.3% Triton X-100, and stained with Apollo fluorescent dyes. A total of 5 mg/mL of 4′,6-diamidino-2-phenylindole (DAPI) was used to stain the cell nuclei for 10 min. The number of Edu-positive cells was counted under a fluorescent microscope in five random fields. All assays were independently performed three times.

### Subcutaneous tumorigenesis in nude mice

A total of 5 × 10^6^ logarithmically growing MCF-7 breast cancer cells carrying the ShRNA-NAP1L1 and their corresponding control cells were injected into the fourth pair of nude mice breast fat pads (BALB/C, nu/nu, female 4 weeks-old, one group = 5). The animals were fed an autoclaved laboratory rodent diet. At 24 days post-injection, the mice were sacrificed with barbiturate at a concentration of 100 mg/kg. Subsequently, the tumor tissues were excised and weighed. All animal studies were conducted in accordance with the principles and procedures outlined in the Southern Medical University Guide for the Care and Use of Animals.

### Western blot analysis

Total protein was extracted from cells using radioimmunoprecipitation assay (RIPA) lysis buffer (CoWin Biosciences, Beijing, China). Total protein concentration was quantified using the bicinchoninic acid (BCA) method (TIANGEN biotech, Beijing, China). The extracted 30 μg proteins were separated by 10% SDS‐PAGE and further transferred onto polyvinylidene fluoride (PVDF) membranes (Millipore, Bedford). The PVDF membranes blocked with 5% skimmed milk were diluted in 5% BSA (BioFroxx, Germany) for 1 h at room temperature. Antibodies including NAP1L1 (1:1000; Proteintech, Rosemont, USA), CCND1 (1:1000; Proteintech, Rosemont, USA), HDGF (1:1000; Proteintech, Rosemont, USA), Caspase3 (1:500; Proteintech, Rosemont, USA), Caspase9 (1:500; Proteintech, Rosemont, USA), and c-Jun (1:1000; Proteintech, Rosemont, USA) were used in the Western blot assays based on the manufacturer’s instructions (Additional file 4: Table S3). Detection was performed using the enhanced chemiluminescence (ECL) Plus Western blotting detection reagents (Millipore, USA). The specific protein expression levels of the blots were normalized to GAPDH (1:1000; Bioworld, Nanjing, China).

### Coimmunoprecipitation (Co-IP) assay

Co-IP was carried out using the Pierce Co-IP Kit (Thermos Scientific, USA) following the manufacturer’s instructions. The total proteins were extracted and quantified. A total of 3000 µg of specific protein was incubated with normaanti-NAP1L1 (Abcam), anti-HDGF (Proteintech), anti-c-Jun (Proteintech), and anti-IgG antibodies for 12 h at 4 ℃ (Additional file 4: Table S3). The beads were washed, eluted in a sample buffer, and boiled for 10 min at 100 ℃. The immune complexes were subjected to Coomassie Brilliant Blue staining and Western blot analysis. Anti-IgG was used as a negative control.

### Confocal microscopy

The breast cancer cells were cultured overnight (2 × 10^5^ /well) before they were fixed with 4% paraformaldehyde and permeabilized with 0.5% Triton X-100 at room temperature. The cells were incubated with anti-NAP1L1, anti-HDGF, and anti-c-Jun antibodies for 1 h at room temperature. After incubation for half an hour at 37 ℃ with a secondary antibody, coverslips were mounted onto the slides with a mounting solution containing 0.2 mg/mL DAPI. The images were captured by laser scanning confocal microscopy (Zeiss LSM 800).

### Statistical analysis

Statistical analyses were carried out using the SPSS 20.0 statistical software package (SPSS, Chicago, IL, USA). Data are shown as the mean ± SD from at least three independent experiments. Two-tailed Student’s t-test was applied for comparisons between groups. Survival analysis was performed using the Kaplan–Meier method. All statistical tests were two-sided; single, double, and triple asterisks indicate statistical significance (*P < 0.05, **P < 0.01, and ***P < 0.001).

## Results

### NAP1L1 overexpression promotes cell proliferation in breast cancer cells

**Fig. 1:**
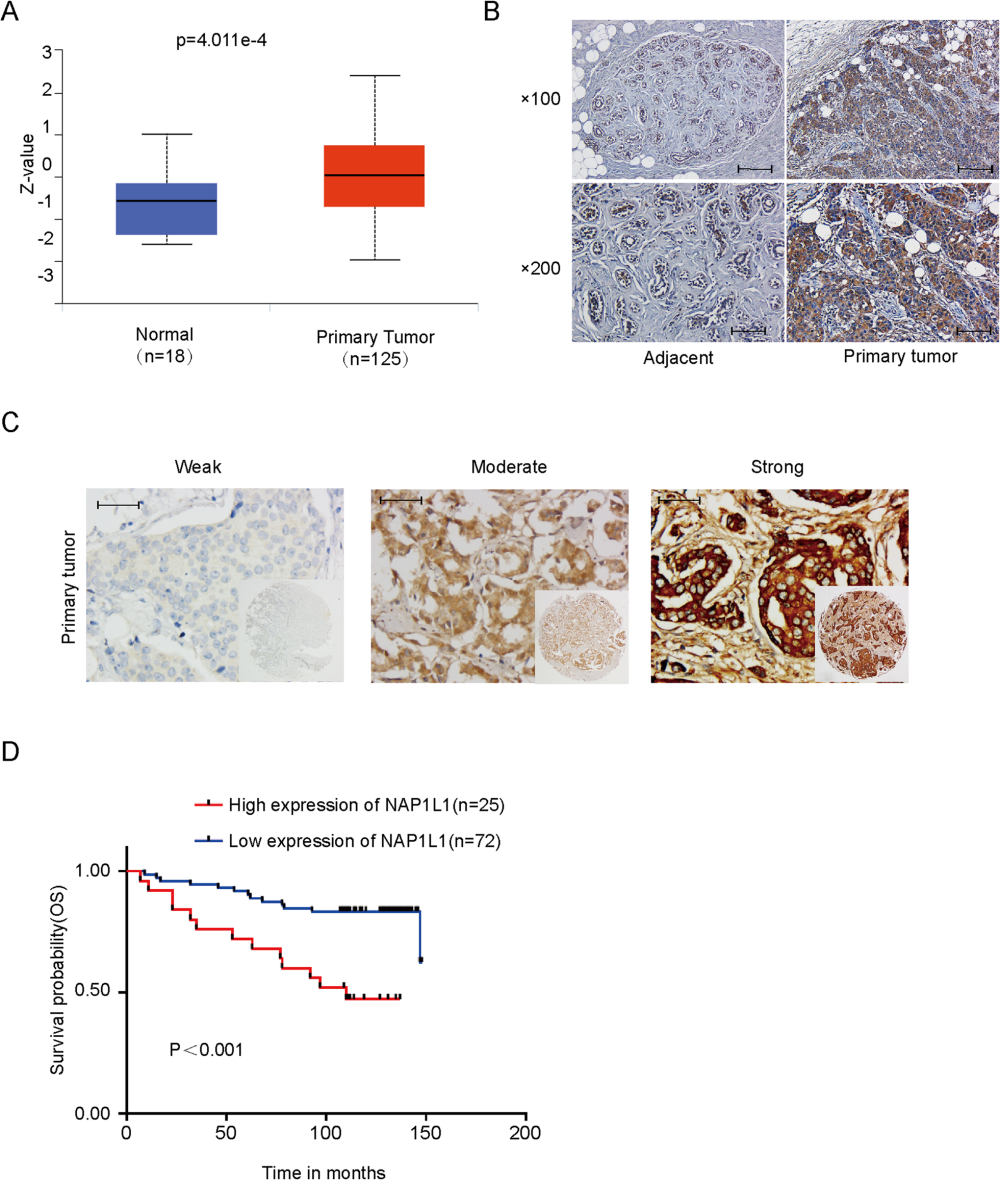
Expression of NAP1L1 in breast cancer. **A** Upregulated NAP1L1 protein expression in breast cancer in CPTAC (http://ualcan.path.uab.edu/) (Z-values represent standard deviations from the median across samples for the given cancer type. The Log2 spectral count ratios obtained from CPTAC were first normalized within each sample profile and then normalized across samples). **B** Upregulated NAP1L1 protein levels were measured via immunohistochemical staining in breast cancer compared to paracarcinoma tissues (97 breast cancer tissue samples and 10 paracarcinoma tissue samples,100 × visual field scale bar: 200 μm 200 × visual field scale bar: 100 μm). **C** NAP1L1 expression performed in TMA (97 breast cancer tissue samples and 10 paracarcinoma tissue samples, scale bar: 50 μm). **D** Kaplan–Meier survival analysis for the overall survival in TMA performed NAP1L1 expression (25 high expression of NAP1L1 samples and 72 low expression of NAP1L1 samples)

To explore the possible role of NAP1L1 in breast cancer, we first examined NAP1L1 expression and its correlation with clinical feature and survival prognosis in breast cancer. According to the analysis of Clinical Proteomic Tumor Analysis Consortium (CPTAC) database, NAP1L1 protein was upregulated (Fig. 1A). Subsequently, A tissue microarray (TMA) containing 97 breast cancer tissue samples and 10 paracarcinoma tissues was used to perform NAP1L1 expression level, and cell staining scores were used to determine low and high expression. We confirmed the upregulated protein levels by immunohistochemistry assay on clinic human breast cancer tissue sections (Fig. 1B, C; Table 1) compared to normal breast tissues.

**Table 1:** The expression of NAP1L1 in breast cancer and adjacent tissues

Group	Case(n)	NAP1L1 expression		P-value^*^
		Low	High	
Breast cancer	97	72 (74.2%)	25 (25.8%)	p < 0.001
Adjacent	10	9 (90%)	1 (10%)	

p-value was determined by a χ^2^-test

Subsequently, we explored the correlation of NAP1L1 protein expression with survival prognosis and clinical features. The data showed that overexpressed NAP1L1 as an unfavorable factor that reduced the overall survival time of breast cancer patients (Fig. 1D). Then, the clinical significance of NAP1L1 expression was assessed (Table 2). Features associated with the survival in univariate Cox regression analysis were tumor scale (0.031), HER-2 (P = 0.014), NAP1L1 expression (P = 0.001), and PR (P = 0.039). Furthermore, multivariate Cox regression analysis indicated that the high NAP1L1 expression level predicted poor survival compared with low NAP1L1 level (Table 3). These data indicate the increased NAP1L1 level as a tumor promoter in breast cancer.

**Table 2:** Correlation of NAP1L1 expression with clinicopathological characteristics of patients with Breast cancer

Characteristics	n	NAP1L1 expression		
		High	Low	p
Age(year)				
< 50	43	15 (34.9%)	28 (65.1%)	0.067
≥ 50	54	10 (18.5%)	44 (81.5%)	
Clinical stage				
I	8	1 (12.5%)	7 (87.5%)	0.025
II	53	9 (17.0%)	44 (83.0%)	
III	36	15 (41.7%)	21 (58.3%)	
Tumor scale				
≤ 3 cm	53	13 (24.5%)	40 (75.5%)	0.758
> 3 cm	44	12 (27.3%)	32 (72.7%)	
Histological grade				
I	29	9 (31.0%)	20 (69.0%)	0.439
II–III	68	16 (23.5%)	52 (76.5%)	
Vital states				
Die	26	13 (50.0%)	13 (50.0%)	0.001
Alive	71	12 (16.9%)	59 (83.1%)	
ER				
Negative	36	12 (33.3%)	24 (66.7%)	0.191
Positive	61	13 (21.3%)	48 (78.7%)	
PR				
Negative	40	14 (35.0%)	26 (65.0%)	0.082
Positive	57	11 (19.3%)	46 (80.7%)	
HER2				
Negative	60	15 (25.0%)	45 (75.0%)	0.825
Positive	37	10 (27.0%)	27 (73.0%)	
Lymph metastasis				
No	32	6 (18.8%)	26 (81.2%)	0.267
Yes	65	19 (29.2%)	46 (70.8%)

**Table 3:** Summary of univariate and multivariate Cox regression analysis

Parameter	Univariate analysis			Multivariate analysis		
	Hazard ratio	95.0% CI	p	Hazard ratio	95.0% CI	p
Age (year)	1.216	0.551–2.687	0.628			
Clinical stage	23.370	0.064–8597.978	0.296			
Tumor scale	1.000	0.454–2.202	0.999			
Histological grade	0.832	0.395–1.929	0.668			
ER	0.548	0.254–1.185	0.126			
PR	0.439	0.201–0.958	0.039	0.554	0.249–1.232	0.147
HER2	1.553	0.717–3.361	0.264			
Lymph metastasis	0.989	0.441–2.221	0.979			
NAP1L1 expression	3.851	1.754–8.455	0.001	3.381	1.512–7.559	0.003

### Downregulated NAP1L1 inhibits cell proliferation

**Fig. 2:**
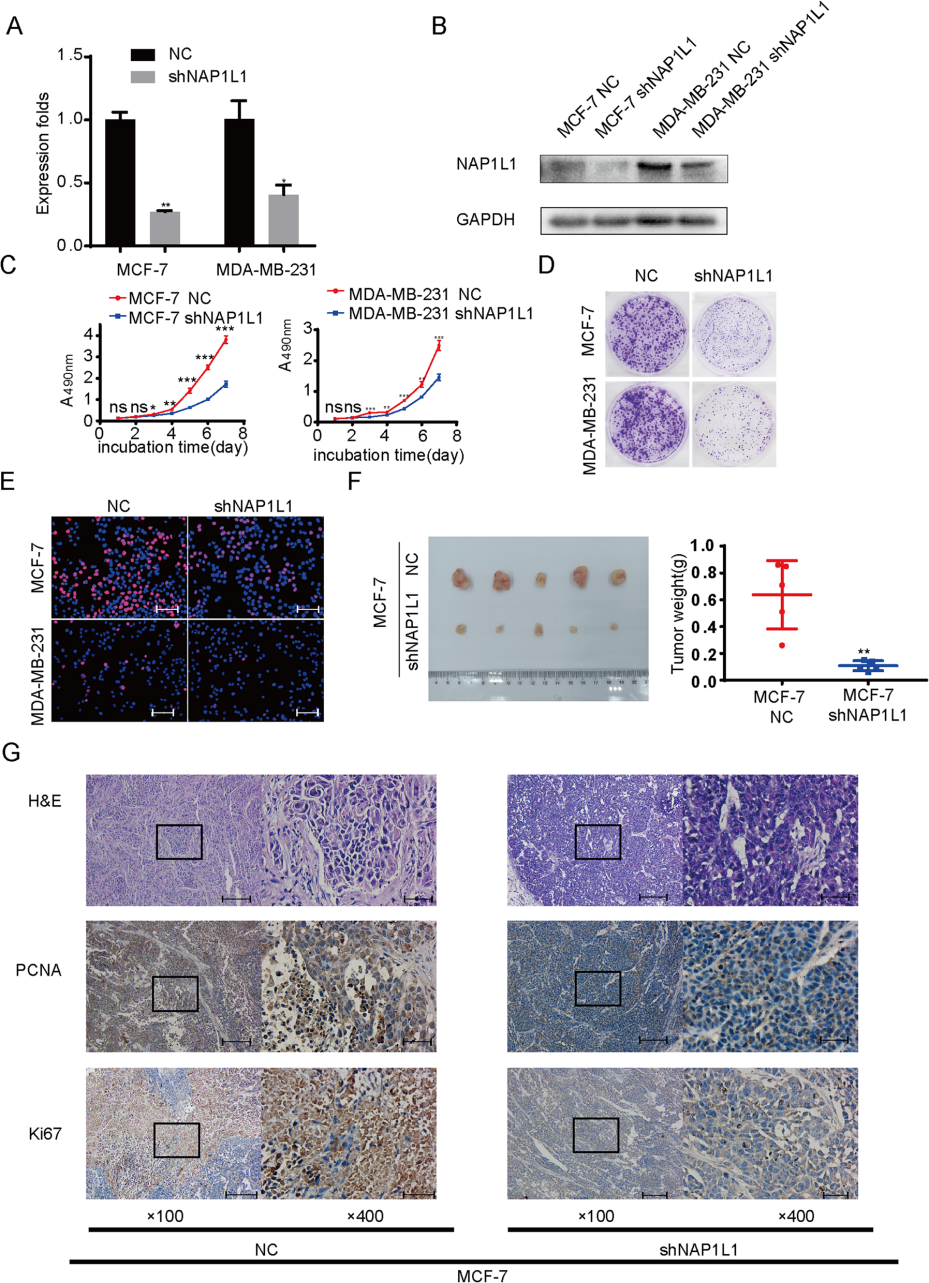
Stably downregulated NAP1L1 attenuated breast cancer cell proliferation. **A** RT-qPCR showed that NAP1L1 mRNA expression was upregulated after breast cancer cells were infected with shRNA-NAP1L1 lentivirus compared to negative control (NC) lentivirus. **B** Western blot analysis showed that the protein level of NAP1L1 was upregulated after the breast cancer cells were infected with shRNA-NAP1L1 lentivirus compared to NC lentivirus. **C** MTT assay indicated that shRNA-NAP1L1 inhibited the cell proliferation in breast cancer cells. **D** Knockdown of NAP1L1 expression suppressed the plate clone formation in vitro. **E** Downregulation of NAP1L1 suppresses EdU staining of breast cancer (scale bar: 200 μm). The data are presented as mean ± s.d. from three independent experiments. *P < 0.05 vs. control; **P < 0.01; ***P < 0.001. **F** Xenograft tumor of nude mice showed that the average weight and volume of tumors decreased in shRNA-NAP1L1 group compared with the negative control group (n = 5 per group). **G** Expression levels of PCNA and Ki-67 were measured via IHC staining in the xenograft tumor of nude mice (100 × visual field scale bar: 200 μm, 400e bar: bar: 50 μm). The data are presented as mean ± s.d. from three independent experiments. *P < 0.05 vs. control; **P < 0.01; ***P < 0.001

**Fig. 3:**
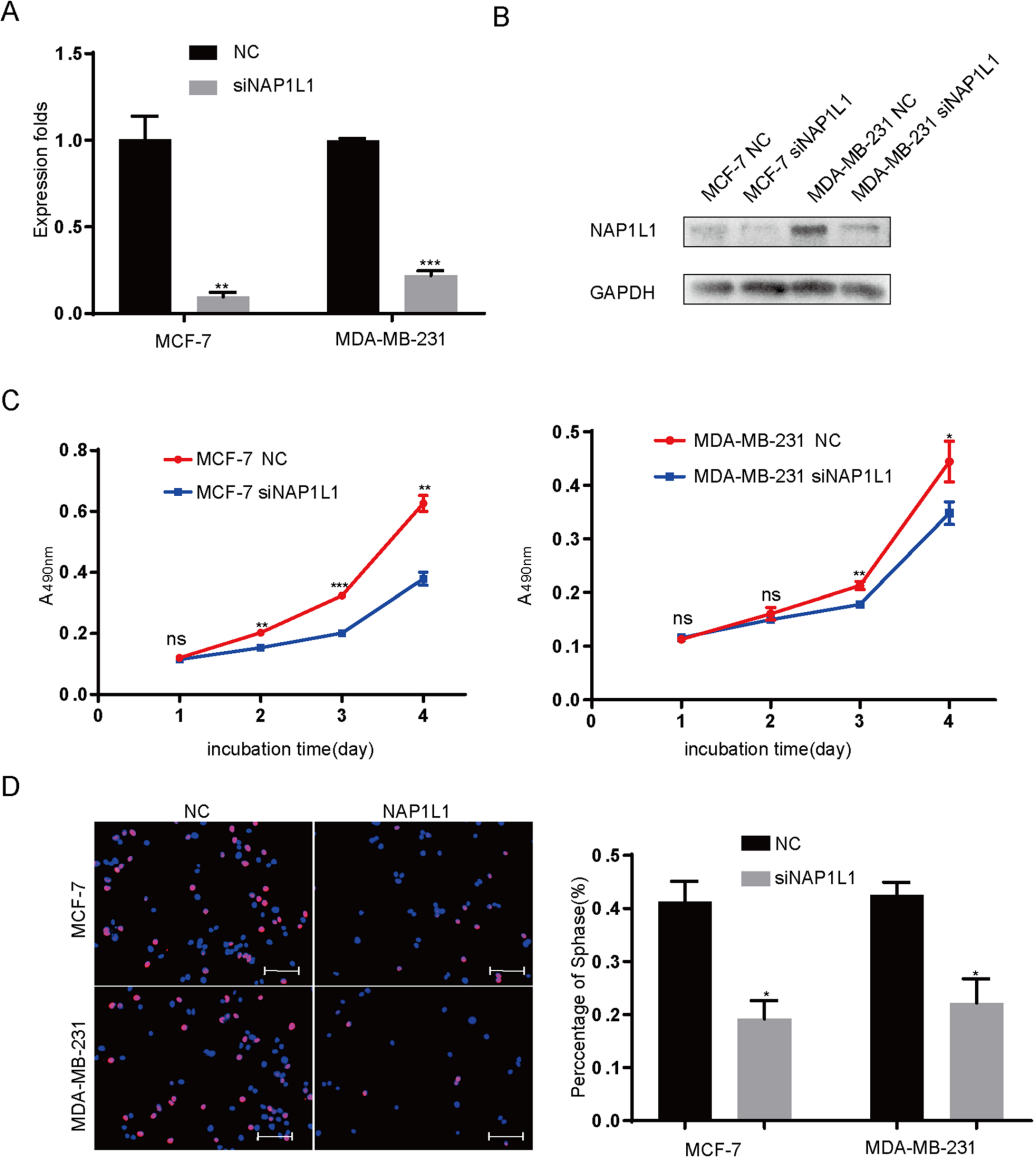
SiRNA-NAP1L1 reduces cell proliferation in vitro. **A** RT-qPCR showed that NAP1L1 mRNA expression was upregulated after the breast cancer cells were transfected with siRNA-NAP1L1 lentivirus compared to NC siRNA-control. **B** Western blot analysis showed that the protein level of NAP1L1 was upregulated after the breast cancer cells were transfected with siRNA-NAP1L1 lentivirus compared to NC siRNA-control. **C** MTT assays showed that the inhibition of NAP1L1 reduces cell proliferation in vitro in breast cancer MCF-7 and MDA-MB-231 cell lines. **D** Downregulation of NAP1L1 suppressed the EdU staining of breast cancer cells in vitro (scale bar: 200 μm). Scrambled siRNA was as an control for siRNA. The data are presented as mean ± s.d. from three independent experiments. *P < 0.05 vs. control; **P < 0.01; ***P < 0.001

To analyze the action of NAP1L1 in breast cancer, lentivirus-carrying shRNA-NAP1L1 was infected into MCF-7 and MDA-MB-231 cells. The transfection efficiency was first analyzed by a real-time quantitative PCR (qRT-PCR) analysis and Western Blot analysis. The data show that the NAP1L1 mRNA and protein levels were significantly downregulated in NAP1L1-knocking down breast cancer cells (Figs. 2A, 3B). In addition, the results of MTT (Fig. 2C), plate clone (Fig. 2D) and EdU staining (Fig. 2E) assays confirm that shNAP1L1 inhibits cell growth and transition of cells into S phase. Here, we also examined the protein expression of Caspase3 and Caspase9 and found their upregulated expression in NAP1L1-knocking down breast cancer cells. This data suggest that NAP1L1-knocking down induces the cytotoxic effect on breast cancer cells (Additional file 1: Fig. S1). Furthermore, an in vivo study was carried out. The average weight and volume of tumors significantly decreased in the *xe*nograft mice after the injection of *NAP1L1-*decreasing breast cancer *c*ells compared with the negative control group (Fig. 2F). Then, the ki-67 and PCNA expressions in xenograft tumors of nude mice were detected. The data show that the ki-67 and PCNA expression of the group injected with shRNA-NAP1L1 was significantly lower than that of the mock xenograft group (Fig. 2G). These data indicate that NAP1L1 functions as a potential oncogene in breast cancer.

### SiRNA-NAP1L1 reduces cell proliferation in vitro

To further confirm the role of NAP1L1 in breast cancer, siRNAs were used to transiently reduce the NAP1L1 mRNA and protein expression levels in breast cancer cells (Fig. 3A, B). Then, MTT and EdU assays showed that the reduced NAP1L1 protein level significantly decreased the cell growth (Fig. 3C). EdU staining assay confirms the results obtained from MTT assay (Fig. 3D). These results further support NAP1L1 as a potential oncogene in breast cancer.

### NAP1L1 interacts with HDGF

**Fig. 4:**
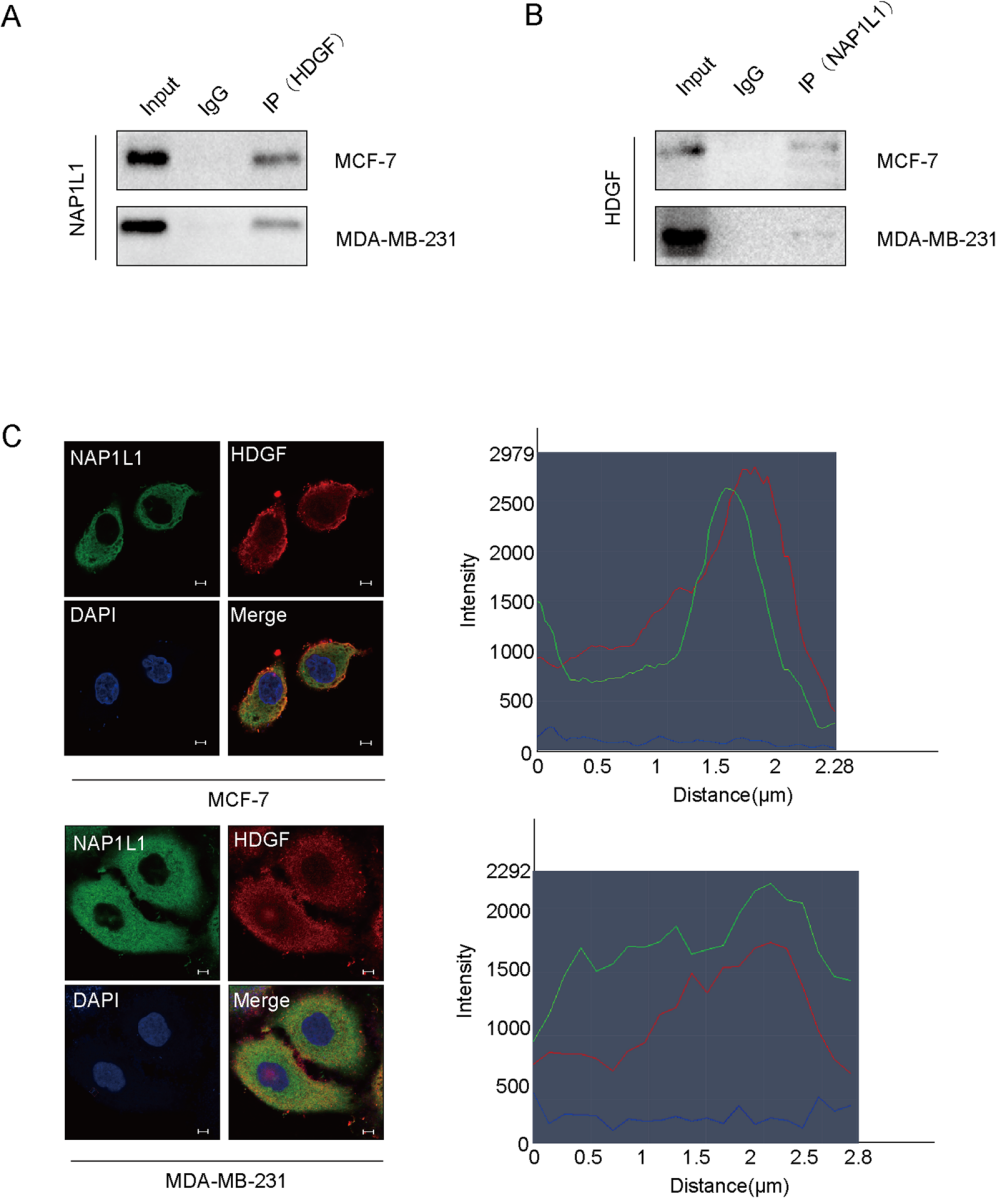
NAP1L1 interacts with HDGF. **A**, **B** Co-IP assay was performed to identify the interaction of NAP1L1 with HDGF. **C** Immunofluorescence of breast cancer cells showed subcellular localization of NAP1L1 (green) and HDGF (red) by confocal microscopy. DAPI (blue) figure showed nucleus. Merge figure showed yellow dots representing colocalization of NAP1L1 and HDGF in the cytoplasm. (scale bar: 5 μm). The data are obtained from three independent experiments

Here, we tried to use CoIP to confirm the interaction of HDGF with NAP1L1 in breast cancer. Endogenous Co-IP assay confirmed that NAP1L1 bound to HDGF (Fig. 4A, B). The confocal microscopic images showed the colocalization of NAP1L1 and HDGF in the cytoplasm of breast cancer cells (Fig. 4C). These results demonstrate the interaction of NAP1P1 with HDGF in breast cancer.

### HDGF recruits c-Jun

**Fig. 5:**
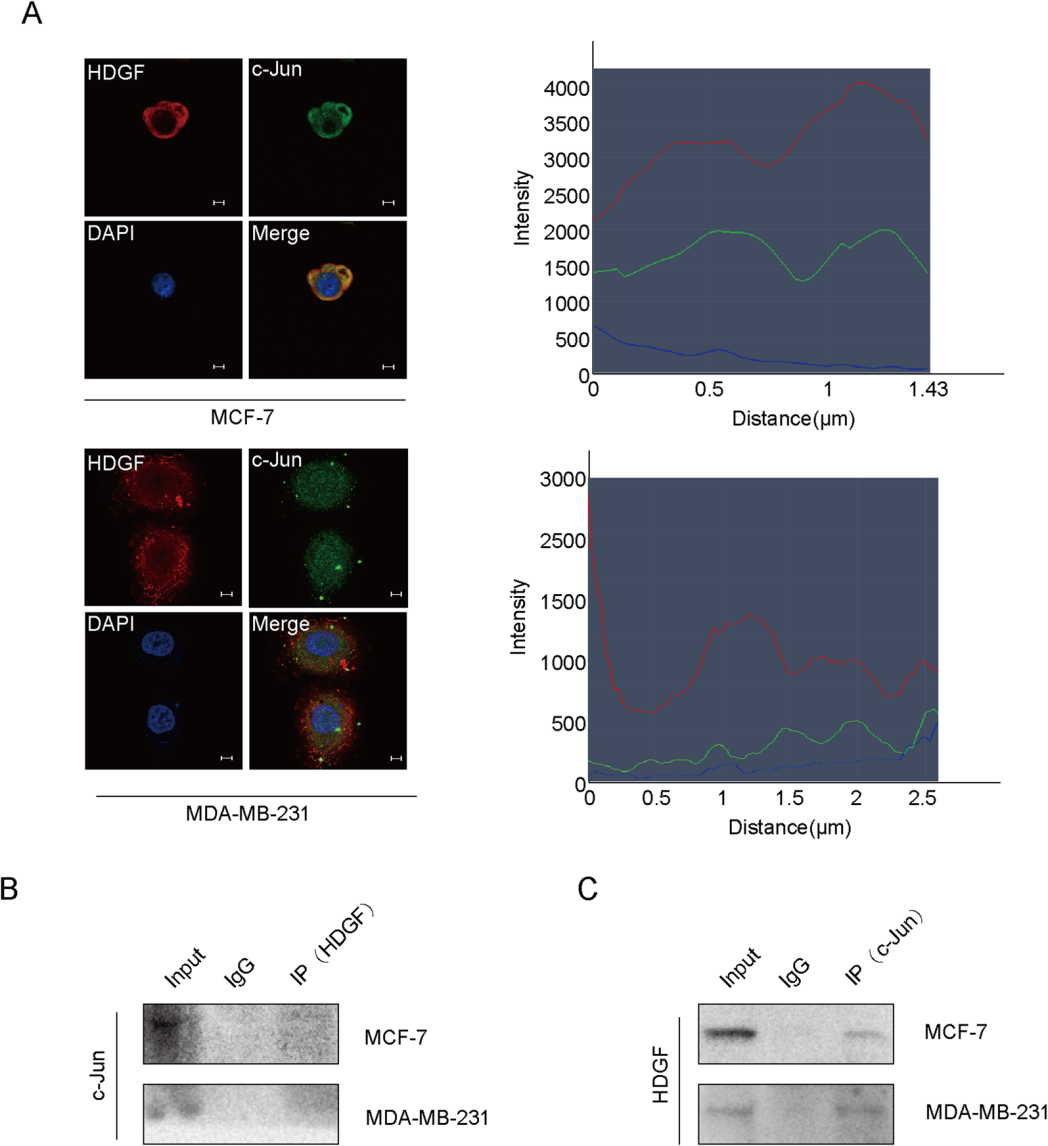
HDGF recruits c-Jun. **A** Immunofluorescence of breast cancer cells showed subcellular localization of HDGF (red) and c-Jun (green) by confocal microscopy. DAPI (blue) figure showed nucleus. Merge figure showed yellow dots representing colocalization of HDGF and c-Jun in the cytoplasm and nucleus. (scale bar: 5 μm). **B**, **C** Co-IP assay was performed to identify the interaction of HDGF with c-Jun. The data are obtained from three independent experiments

C-Jun was predicted as a potential interactor of HDGF based on the BioGrid database (https://thebiogrid.org/109928/summary/homo-sapiens/jun.html). In addition, the confocal microscopic images showed that HDGF interacts with c-Jun in the cytoplasm and nucleus (Fig. 5A). The Co-IP assay shows the interaction between c-Jun and HDGF in breast cancer (Fig. 5B, C). These results demonstrate c-JUN as an interactor of HDGF in breast cancer.

### Transfecting HDGF increases c-Jun/CCND1 signal and restores cell proliferation in NAP1L1-suppressing breast cancer cells

**Fig. 6:**
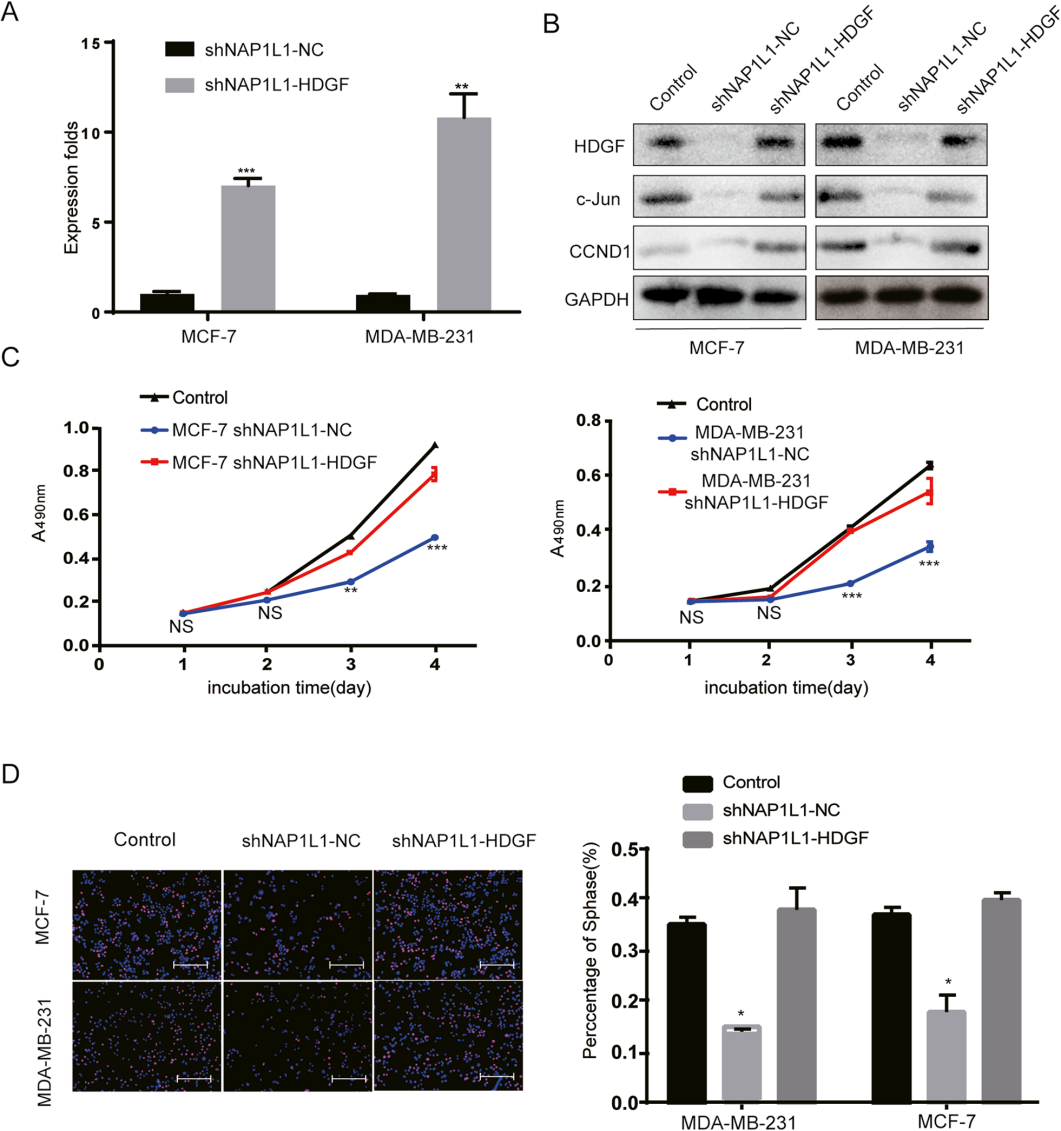
Transfecting HDGF restores the cell proliferation in NAP1L1-suppressing breast cancer cells through c-Jun/CCND1 signal. **A** NAP1L1, HDGF, c-Jun, and CCND1 protein levels were evaluated in NC, shNAP1L1, and HDGF-shNAP1L1 breast cancer cells. **B** RT-qPCR data was used to measure the HDGF expression after restoring HDGF in shNAP1L1 breast cancer cells. **C** MTT assays indicated that transfecting HDGF restores the cell growth of breast cancer. **D** Transfecting HDGF restores EdU staining of breast cancer (scale bar: 200 μm). The data are presented as mean ± s.d. from three independent experiments. *P < 0.05 vs. control; **P < 0.01; ***P < 0.001

To confirm HDGF as a downstream of NAP1L1 to participate in NAP1L1-induced breast cancer pathogenesis, HDGF cDNA plasmid was transfected to NAP1L1-suppressing cells. The upregulated efficiency of HDGF was verified by qRT-PCR and Western blot analysis (Fig. 6A, B). According to our observation in vitro, the ability of cell proliferation (Fig. 6C) and EdU staining (Fig. 6D) was obviously restored. Western blot assay indicated that the intensity of c-Jun/CCND1 signal significantly increased (Fig. 6B). These data demonstrate that HDGF mediated NAP1L1-promoted breast cancer development.

### C-Jun transfection enhances CCND1 signal and restores cell proliferation in NAP1L1-suppressing breast cancer cells

**Fig. 7:**
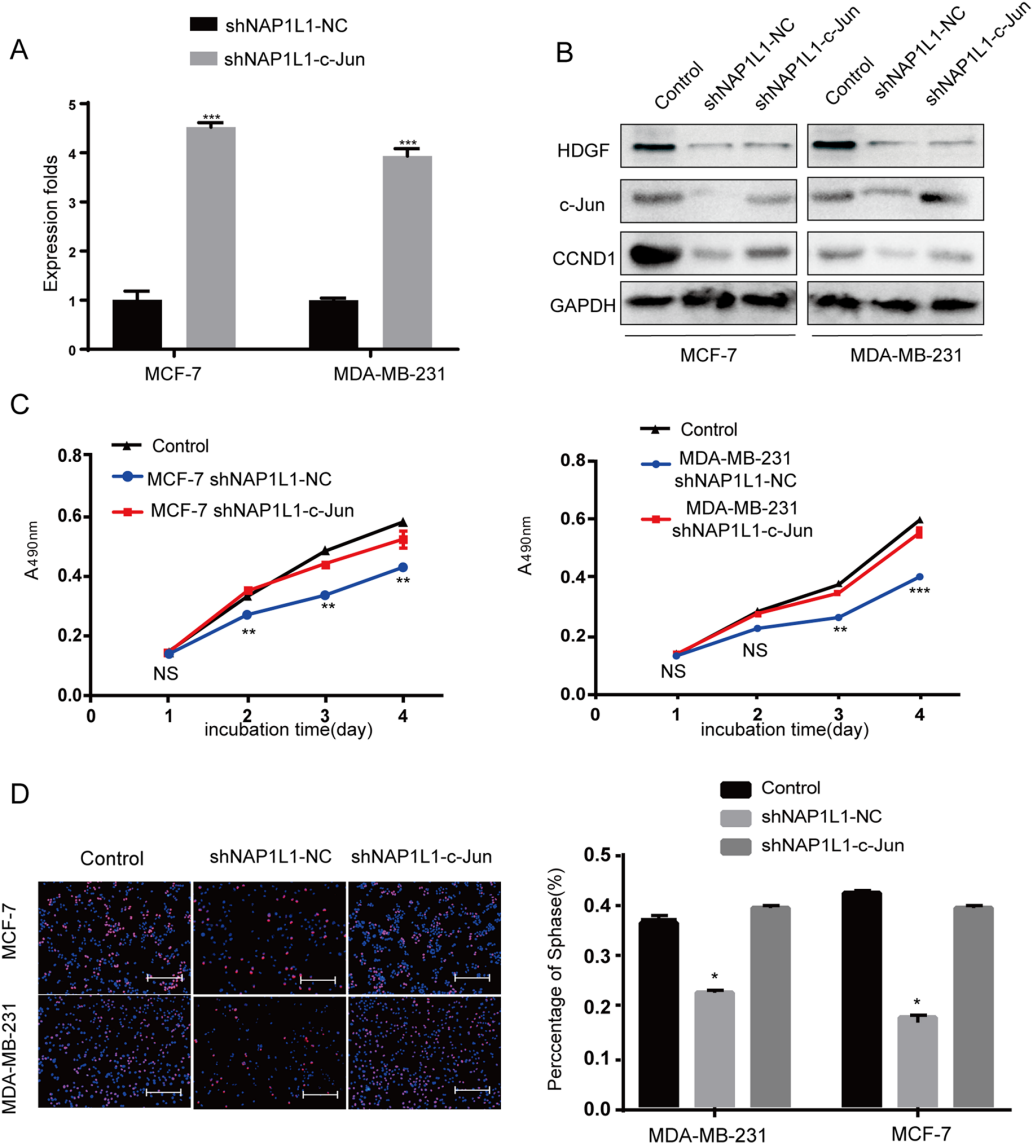
Transfecting c-Jun increases the CCND1 signal and restores the cell growth in NAP1L1-suppressing breast cancer cells. **A** RT-qPCR was used to measure the c-Jun expression after restoring c-Jun in NC, shNAP1L1, and c-Jun-shNAP1L1 of breast cancer cells. **B** C-Jun and CCND1 protein levels were measured when c-Jun was transfected in shNAP1L1-breast cancer cells by western blot analysis. **C** MTT assays showed that transfecting c-Jun restores the cell proliferation of breast cancer cells. D: EdU assay showed that transfecting c-Jun restores the cell cycle progression (scale bar: 200 μm). The data are presented as mean ± s.d. from three independent experiments. *P < 0.05 vs. control; **P < 0.01; ***P < 0.001

To confirm c-JUN to participate in NAP1L1-induced breast cancer pathogenesis, c-Jun cDNA plasmid was transfected to NAP1L1-suppressing cells. Using qRT-PCR and western blot (Fig. 7A, B), significant mRNA and protein upregulation of c-Jun and CCND1 was observed. Furthermore, the ability of cell proliferation (Fig. 6C) and EdU staining (Fig. 6D) was restored in vitro in NAP1L1-suppressing breast cancer cells. These results demonstrate that c-JUN mediated NAP1L1-promoted breast cancer development.

## Discussion

In previous studies, NAP1L1 is reported to correlate with tumor pathogenesis. Chen et al. observed that PRDM8 suppresses the occurrence and development of hepatocellular carcinoma pathogenesis by targeting NAP1L1 to suppress phosphatidylinositol 3-hydroxy kinase (PI3K)/protein kinase B (PKB)/mammalian (or mechanistic) target of rapamycin (mTOR) signal. Zhai et al. found that miR-532-5p suppresses renal cancer cell proliferation by disrupting the E26 transformation specific-1 (ETS1)-mediated positive feedback loop with the KRAS-NAP1L1/P- extracellular signal-regulated kinase (ERK) axis [11, 12]. In addition, NAP1L1 knockdown attenuates p65 binding to the antiapoptotic Mcl-1 gene promoter and reduces its expression, which finally induced cell apoptosis in tumor cells [18]. It also epigenetically promotes cell proliferation through the negative regulation of p57 (Kip2) promoter methylation in pancreatic neuroendocrine neoplasm. NAPIL1 also mediated LncRNA CDKN2B-AS1 to promote tumor growth and metastasis of human hepatocellular carcinoma [13, 14]. These mechanistic studies demonstrated that NAP1L1 as a tumor promoter participated in tumor pathogenesis. However, the role and molecular basis of NAP1L1 in breast cancer have never been documented.

To investigate the possible role of NAP1L1 in breast cancer, we first observed that the expression of NAP1L1 protein was significantly upregulated in breast cancer tissues compared to normal breast tissues based on the CPTAC database. Further, immunochemistry was used to detect the expression of NAP1L1 protein in breast cancer tissues and normal breast tissues. The data show a significant elevation of NAP1L1 protein expression in breast cancer tissues. Furthermore, an increase in NAP1L1 protein expression was found to be a predictor for poor survival prognosis in the high NAP1L1 expression group compared with the low expression group in breast cancer patients. Finally, multivariable analysis demonstrated that increased NAP1L1 expression is an independent prognosis marker for the overall survival in breast cancer. These data are consistent with the reported data on lung adenocarcinoma, colorectal cancer, and hepatocellular carcinoma (HCC) [8–10], suggesting that NAP1L1 promotes the pathogenesis of breast cancer and might be a potential tumor promoter in breast cancer.

In previous studies, NAP1L1 was reported as a potential oncogene promoting cell growth [10–14, 18]. Here, we also investigated the role of NAP1L1 in breast cancer cell growth. After transiently or stably suppressing NAP1L1 expression in breast cancer cells by respectively using siRNA or lentivirus-mediated shRNA, it was observed that the ability of cell proliferation and EDU staining was significantly reduced in vitro. These data demonstrate that NAP1L1 induced tumor regression is cytostatic. Furthermore, we also observed that knocking down NAP1L1 upregulated the expression of Caspase3 and Caspase9 protein levels, indicating that increased cytotoxicity is also involved in tumor suppression in NAP1L1 knocking down breast cancer cells. In vivo experiment showed that the ability of subcutaneous tumor formation of breast cancer cells significantly decreased after the expression of NAP1L1 was stably inhibited. The abovementioned data further supported NAP1L1 as a potential oncogene in breast cancer. This finding is consistent with the role of NAP1L1 in several tumors [10–14, 18], indicating the importance of NAP1L1 in breast cancer pathogenesis. However, the molecular basis of NAP1L1 in breast cancer is still unclear.

HDGF was originally obtained from the conditioned media of HuH-7 hepatoma cells [19]. It has been widely reported as an oncogenic factor promoting tumor pathogenesis including nonsmall cell lung carcinoma (NSCLC), endometrial cancer, nasopharyngeal carcinoma, breast cancer, and liver cancer in previous studies [20–25]. In a recent study, Wang et al. found that methyltransferase-like 3 (METTL3) mediated m(6)A modification of HDGF mRNA and increased its mRNA stability. The secreted HDGF induced tumor angiogenesis, while nuclear HDGF activated glucose transporter type 4 (GLUT4) and enolase-2 (ENO2) expression, followed by an elevation in glycolysis in gastric cancer cells, which was correlated with subsequent tumor growth and liver metastasis [26]. Interestingly, anti-HDGF antibody treatment has been shown to enhance the antitumor activities of gemcitabine, bevacizumab, and chemotherapy and abolished HDGF-stimulated hypoxia-inducible factor (HIF)-1α, nuclear factor (NF)-κB, and vascular endothelial growth factor (VEGF) protein expression in respective lung cancer and oral cancer, which hinted HDGF as a significant therapy target in tumors [27–29]. Taken together, these data indicated the significance of HDGF in tumor pathogenesis.

Protein–protein interactions (PPIs) are essential for most cell events, therefore, understanding PPIs have become a key issue to understand the cell physiology in tumors [20–33]. In previous studies, HDGF had been shown to combine with DEAD box protein 5 (DDX5) in tumors [23, 34]. In this study, Xiao, a member of our group, carried out an Co-IP assay combined with mass spectrometry to search the potential interaction factors of HDGF in endometrial carcinoma (data not shown. We can provide the original data on request, but they did not publish them). Interestingly, NAP1L1 was screened as one of the candidate interaction proteins of HDGF. Here, NAP1L1 was found to combine with HDGF by Co-IP examination in breast cancer cells. Furthermore, it was confirmed that these two proteins are co-located in the cytoplasm of breast cancer. These data show that HDGF is involved in NAP1L1-induced pathogenesis of breast cancer.

To further explore the molecular basis of NAP1L1 in inducing cell growth via HDGF, BioGrid database was used to predict the interactive proteins of HDGF. Excitingly, c-Jun was predicted as a potential candidate of HDGF. C-Jun is an oncogenic transcription factor stimulating the expression of some genes [33, 35–38], which promotes the occurrence and development of many tumors. In this study, the interaction of HDGF with c-Jun was further confirmed using an endogenous Co-IP assay. Furthermore, HDGF and c-Jun were proved to be co-located in the cytoplasm and nucleus. This result indicated that HDGF recruits c-Jun to participate in breast cancer pathogenesis.

CCND1 is a significant cell cycle promoter inducing cell proliferation in tumors [39–41]. It is also a significant transcription product of c-Jun [42, 43]. To prove whether CCND1 participated in NAP1L1/HDGF/c-Jun signal-induced cell proliferation, HDGF or c-Jun cDNA plasmid was transfected into shNAP1L1-treated breast cancer cells, and it was found that the c-Jun/CCND1 expression level was significantly upregulated in shNAP1L1-treated breast cancer cells. Furthermore, the cell proliferation ability was also restored in NAP1L1-suppressed breast cancer cells. These data demonstrated that HDGF/c-Jun/CCND1 signal positively participated in NAP1L1-induced breast cancer growth.

Taken together, an increased NAP1L1 protein level is an unfavorable outcome for breast cancer patients. It functions as a potential oncogene that interacts with HDGF to recruit c-Jun and thus stimulates CCND1 expression to induce cell cycle transition, finally promoting cell proliferation in breast cancer.

## Supplementary Information

**Additional file 1: Figure S1.** NAP1L1-knocking down mediated in cytotoxic effect participated in inducing breast cancer regression. Western blot analysis showed that the protein level of caspase3 and caspase9 was upregulated in breast cancer cells after infection with shNAP1L1 lentivirus compared to NC lentivirus. The data are obtained from three independent experiments.

**Additional file 2: Table S1.**The primers used in this study.

**Additional file 3: Table S2.** A list of Antibodies used for WB,Co-IP,IF and IHC.

**Additional file 4: Table S3.** Transient and stable disturbance sequences.

## Data Availability

The datasets used during this study are available from the corresponding author on reasonable request.
