# Correction to: NAP1L1 interacts with hepatoma-derived growth factor to recruit c-Jun inducing breast cancer growth

**DOI:** 10.1186/s12935-021-02437-2

**Published:** 2022-01-15

**Authors:** Shu Liu, Yewei Zhang, Shien Cui, Dajiang Song, Bo Li, Qian Chen, Guangyu Yao, Bin Gong

**Affiliations:** 1 Department of Breast Surgery, The Affiliated Hospital of Guizhou Medical University, Guiyang, 550001 Guizhou People’s Republic of China; 2 Guizhou Medical University, Guiyang, Guizhou China; 3 Breast Center, Department of General Surgery, Nanfang Hospital Southern Medical University, Guangzhou, China; 4 Breast Center, Department of General Surgery, Zhongshan City People’s Hospital, Zhongshan, Guangzhou, China; 5 Cancer Center, Integrated Hospital of Traditional Chinese Medicine, Southern Medical University, Guangzhou, China; 6 Department of Oncology Plastic Surgery, Hunan Province Cancer Hospital and The Affiliated Cancer Hospital of Xiangya School of Medicine, Central South University, Changsha, Hunan China

## Correction to: Cancer Cell International (2021) 21:605 https://doi.org/10.1186/s12935-021-02301-3

In this article [1], two pictures incorrectly placed in Figures 4C and 5A; the correct Figs. 4 and 5 should have appeared as shown in this erratum.

**Fig. 4:**
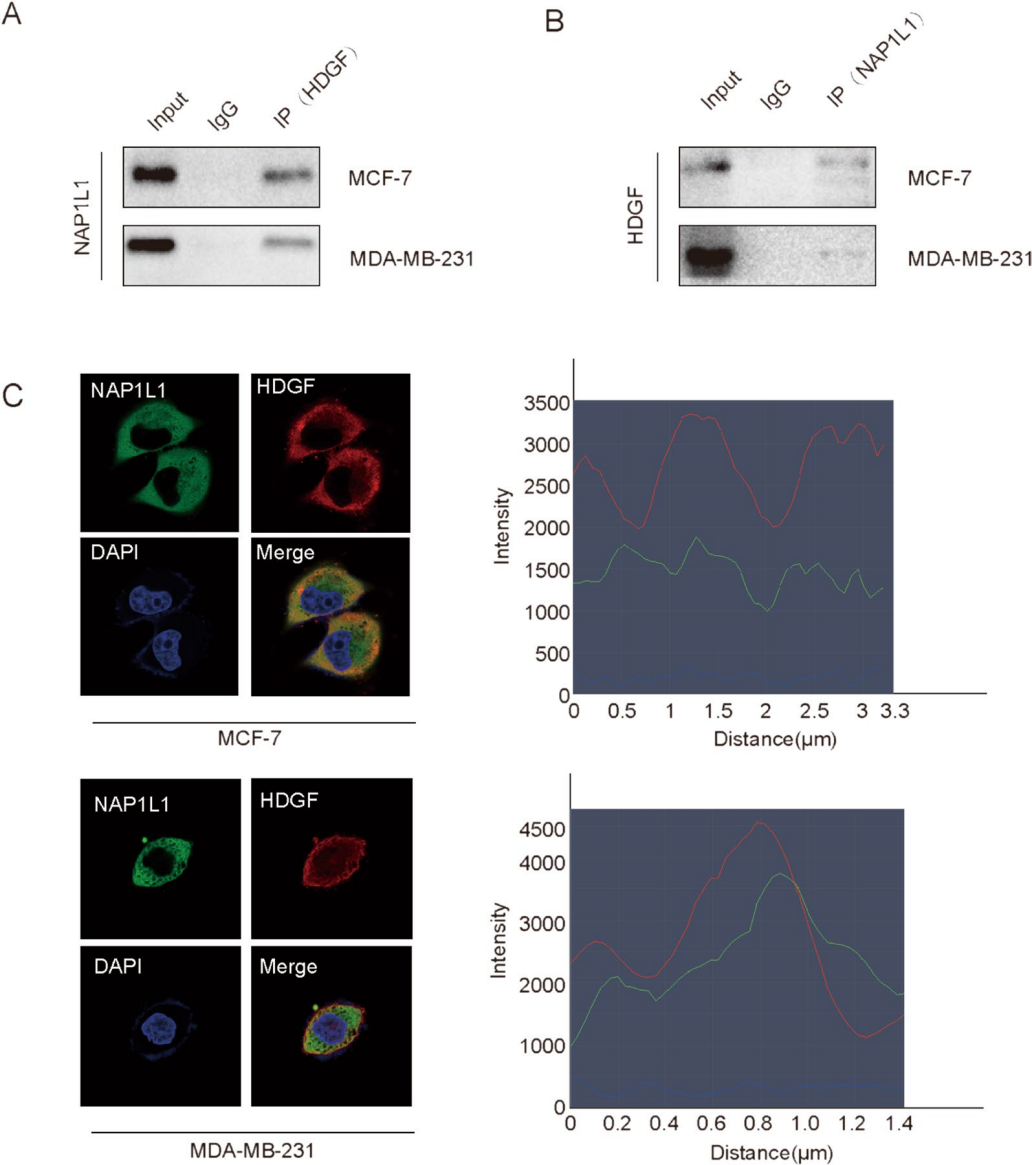
NAP1L1 interacts with HDGF. **A**, **B** Co-IP assay was performed to identify the interaction of NAP1L1 with HDGF. **C** Immunofluorescence of breast cancer cells showed subcellular localization of NAP1L1 (green) and HDGF (red) by confocal microscopy. DAPI (blue) figure showed nucleus. Merge figure showed yellow dots representing colocalization of NAP1L1 and HDGF in the cytoplasm. (scale bar: 5 μm). The data are obtained from three independent experiment

**Fig. 5:**
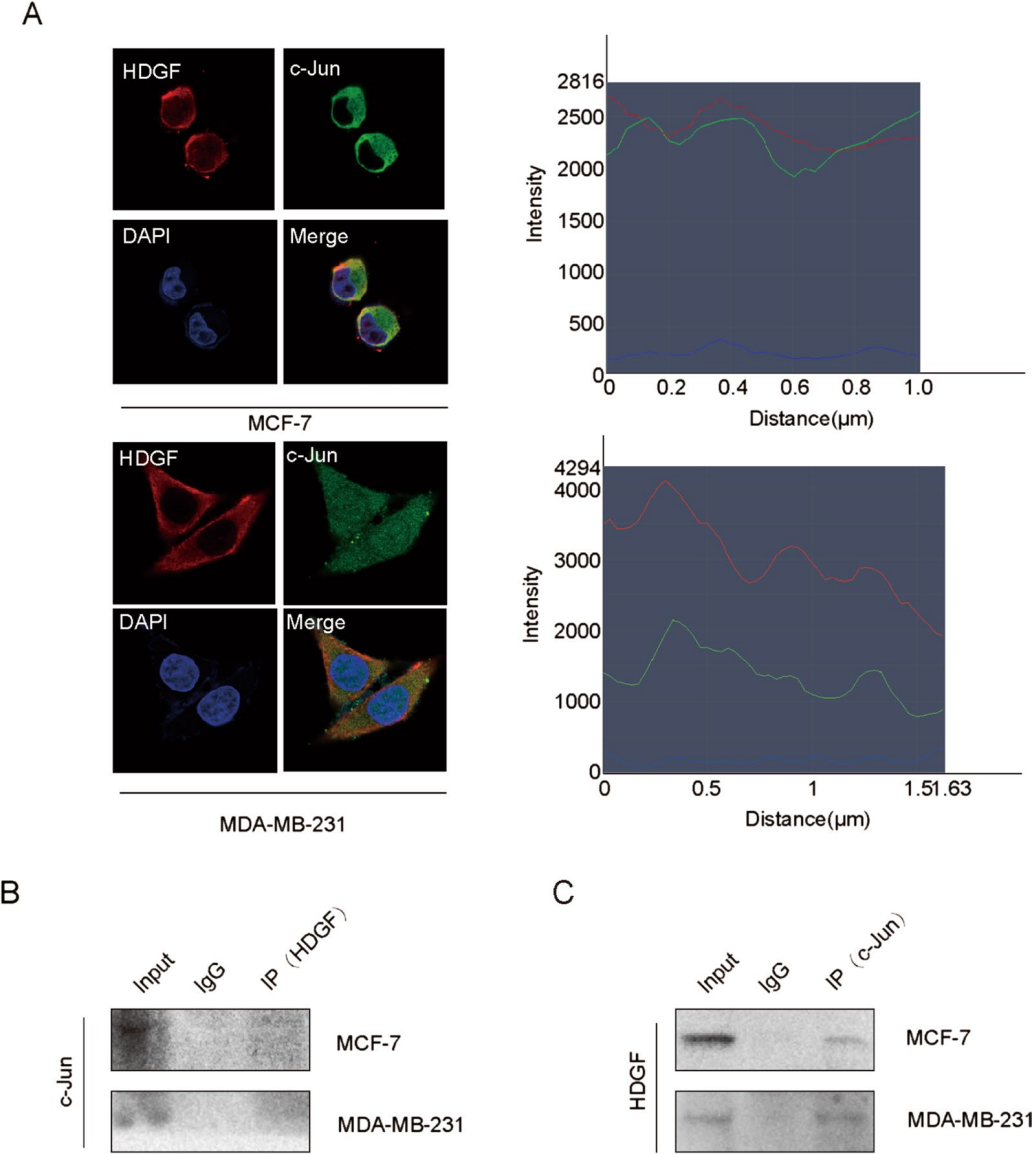
HDGF recruits c-Jun. **A** Immunofluorescence of breast cancer cells showed subcellular localization of HDGF (red) and c-Jun (green) byconfocal microscopy. DAPI (blue) figure showed nucleus. Merge figure showed yellow dots representing colocalization of HDGF and c-Jun in the cytoplasm and nucleus. (scale bar: 5 μm). **B**, **C** Co-IP assay was performed to identify the interaction of HDGF with c-Jun. The data are obtained from three independent experiment
