# Supplementary figures and images for: NAP1L1 interacts with hepatoma-derived growth factor to recruit c-Jun inducing breast cancer growth
Source: Cancer Cell Int. 2021 Nov 13;21:605. doi: 10.1186/s12935-021-02301-3 (PMC8590370; doi:10.1186/s12935-021-02301-3)

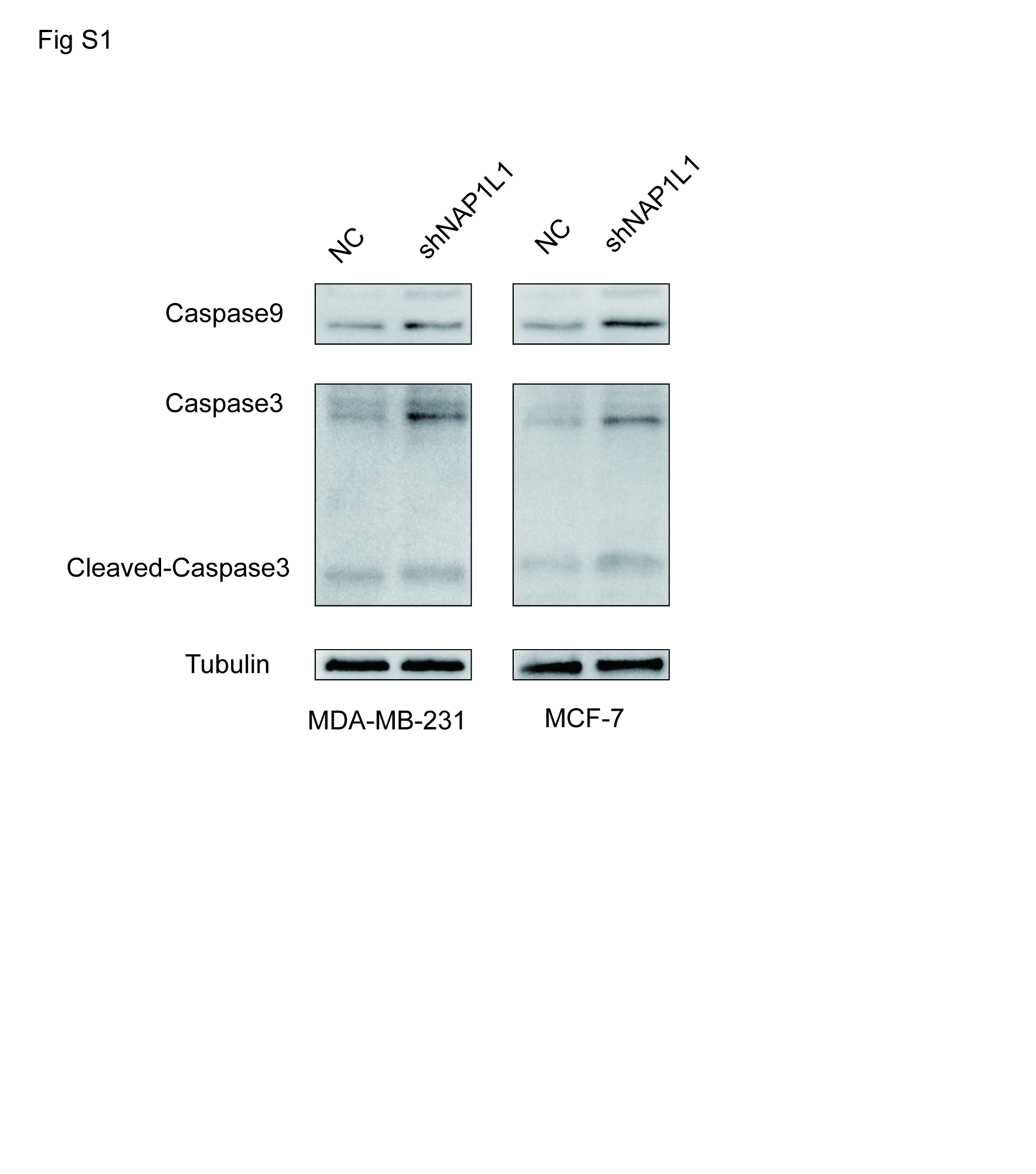

Supplement: Supplementary file 1 — Additional file 1: Figure S1. NAP1L1-knocking down mediated in cytotoxic effect participated in inducing breast cancer regression. Western blot analysis showed that the protein level of caspase3 and caspase9 was upregulated in breast cancer cells after infection with shNAP1L1 lentivirus compared to NC lentivirus. The data are obtained from three independent experiments. [file 12935_2021_2301_MOESM1_ESM.tif]
